# Effectiveness of Yijinjing on cognitive functions in post-stroke patients with mild cognitive impairment: study protocol for a randomized controlled trial

**DOI:** 10.1186/s13063-021-05220-w

**Published:** 2021-04-09

**Authors:** Xin Xue, Xue-Ming Jin, Kai-Liang Luo, Xin-Hao Liu, Li Zhang, Jun Hu

**Affiliations:** 1 School of Rehabilitation Science, Shanghai University of Traditional Chinese Medicine, No. 1200, Cai-Lun Road, Shanghai, China; 2 Shanghai Second Rehabilitation Hospital, Shanghai, China

**Keywords:** Yijinjing, Post-stroke cognitive impairment, Randomized controlled trial, ADL, QOL

## Abstract

**Background:**

Statistics show that every year, 5.4 million people in the world suffer a stroke. Post-stroke cognitive impairment (PSCI) is one of the most common complications after stroke with a rate of 75%, which leads to decreased functions for independent living and reduced quality of life (QOL). Exercise training has been reported to be useful to improve the cognitive functions of post-stroke patients. Yijinjing, a traditional Chinese Qigong exercise characterized by an integration of mind and body in moderate exercise intensity, can improve cognitive functions of PSCI patients. This study aims to explore the feasibility and effectiveness of the Yijinjing exercise in this regard.

**Methods:**

A single-blind, superiority, randomized controlled trial will be employed with evaluations at 3 and 6 months. Seventy-two PSCI patients will be recruited and randomly assigned to the Yijinjing exercise intervention group or the control group (1:1). Participants in the control group will receive routine rehabilitation therapies, including occupational therapy, physical therapy, acupuncture therapy, and health education 5 times a week for 3 months. The intervention group will receive a 12-week routine rehabilitation therapy combined with the Yijinjing exercise intervention for 40 min each session and 3 sessions a week. The primary outcome of cognition will be measured by the Montreal Cognitive Assessment scale (MoCA). Secondary outcomes include executive function, memory function, visuospatial function, sleep quality, gait and motor function, activity of daily living (ADL), and quality of life (QOL).

**Discussion:**

Current evidence has reported the effectiveness of traditional Chinese exercise in improving the post-stroke population’s motor functions. This research is a randomized controlled trial that evaluates traditional Chinese exercise’s effectiveness for PSCI patients. It is expected to expand the traditional Chinese exercise scope and provide a new treatment approach for stroke populations with cognitive impairments.

**Trial registration:**

Chinese Clinical Trial Registry ChiCTR1900026532. Registered on 13 October 2019.

## Background

According to the World Health Organization (WHO), 6.2 million individuals die of stroke every year, while about 5.4 million survive [1]. Studies have shown that 75% of stroke patients live with cognitive impairments [2]. Post-stroke cognitive impairment (PSCI) is one of the most common factors leading to lifelong disabilities after stroke, which significantly impacts the patients’ activities of daily living (ADL) and their quality of life (QOL) [3, 4]. Moreover, healthcare and caregiving costs are high, which poses an economic burden for the healthcare system and families [5].

The symptoms of PSCI mainly include decreased memory, short attention span, decreased executive function, and dyscalculia. Side effects and clinical limitations of pharmacological therapy have been reported [6–8]. Mainstream post-stroke rehabilitation is based on neuroplasticity theory, which believes that repetitive practice after stroke can facilitate new synapses in the brain, thereby improving brain function [9]. As a non-pharmacological approach, exercise therapy has become a feasible strategy to improve PSCI [10, 11]. However, limited motor functions and decreased endurance due to stroke could prevent a post-stroke patient from participating in exercise activities. Low- or moderate-intensity mind-body exercise addresses physical and mental functions, which would be appropriate for this population, especially the older adults. Previous studies have shown that mind-body exercise can potentially improve motor function, psychological function, and ADL of stroke patients [12, 13] . As one of the traditional Chinese exercises, Yijinjing is a moderate-intensity exercise therapy based on traditional Chinese medicine theory. The Yijinjing exercises emphasize the combination of symmetrical physical postures, meditative mind, and breathing techniques in a harmonious manner. The Yijinjing exercise consists of linear movements and requires isolated joint movements. With simplified patterns and directions, Yijinjing is easy to practice with few limitations. The pilot study has demonstrated that the Yijinjing exercise helped improve older people’s physical and psychological outcomes in the community. This study’s primary purpose is to conduct a randomized controlled trial to evaluate the Yijinjing exercise’s effects in improving the cognitive functions of PSCI patients.

### Hypothesis

1. The Yijinjing exercise positively affects the cognitive functions, sleep quality, ADL, and QOL of PSCI patients.
2. The Yijinjing exercise has better outcomes, compared with the routine treatment for PSCI patients.

## Methods/design

### Objectives

The objectives of this study are:

1. To evaluate the Yijinjing exercise’s effectiveness for treating PSCI;
2. To test whether the Yijinjing exercise is more advantageous than routine treatment for PSCI patients;
3. To explore the feasibility of developing the Yijinjing exercise as an approach to improve the cognitive functions of the PSCI population.

### Study design

**Fig. 1:**
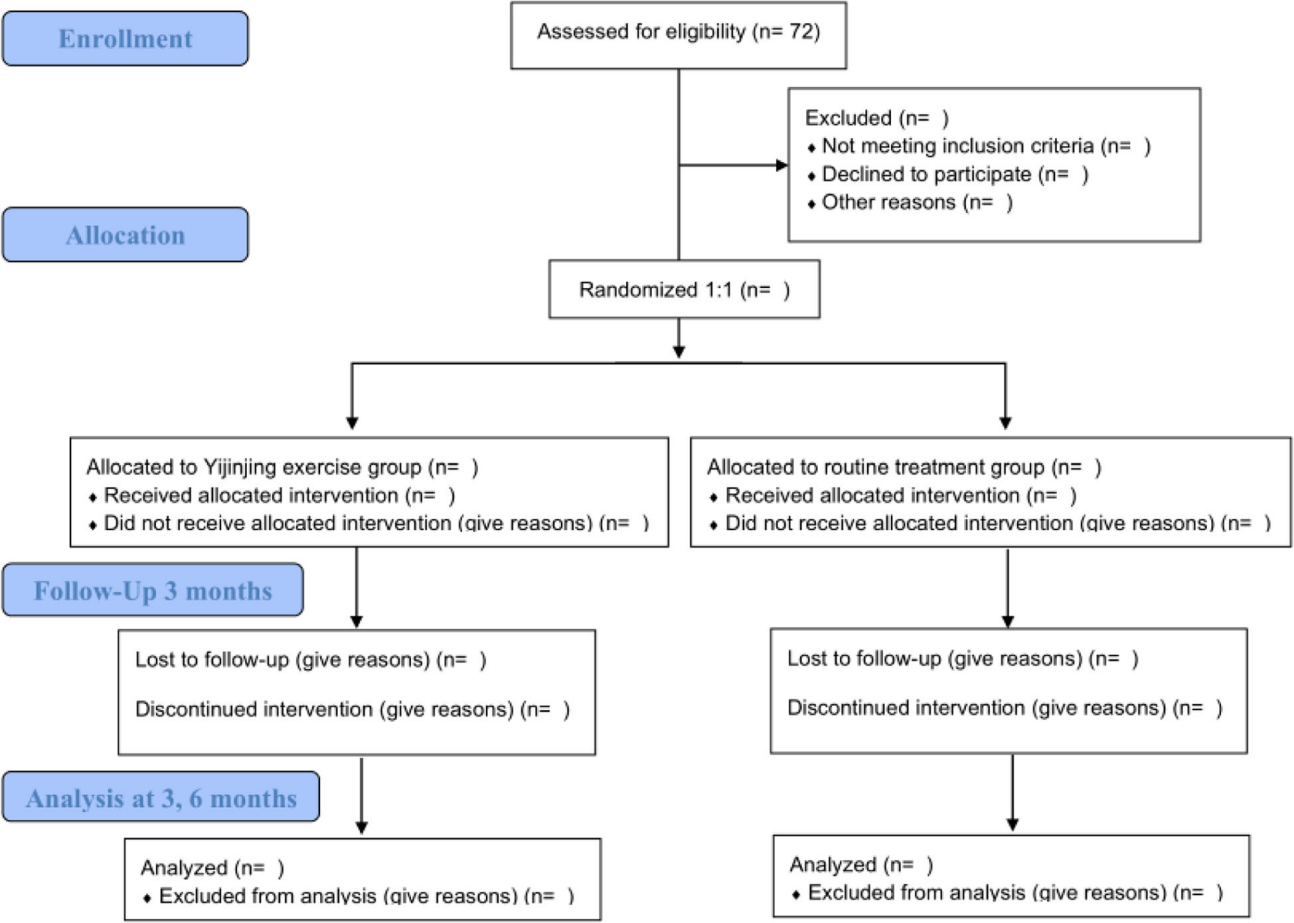
Flow diagram of the study design

**Fig. 2:**
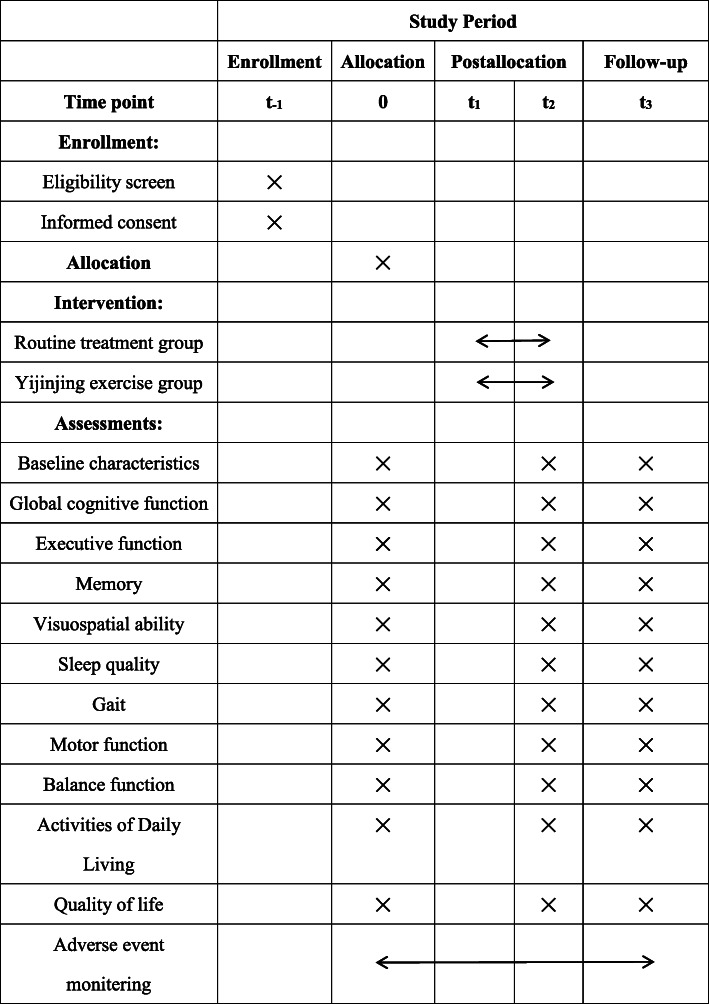
SPIRIT figure showing time points for enrollment, allocation, interventions and assessment. t_-1_ = − 2 week, 0 = baseline, t_1_ = start of treatment week 1, t_2_ = end of treatment week 12, t_3_ = follow-up week 24

A single-blind, superiority, randomized controlled trial (RCT) will be employed to compare the Yijinjing exercise with routine treatment. Seventy-two post-stroke patients with mild cognitive impairment from Yueyang Hospital of Integrated Traditional Chinese and Western Medicine and Shanghai Jinhui Rehabilitation Hospital will be recruited. Before receiving the rehabilitation, the demographic information, such as name, gender, age, educational background, and medical history of all participants will be collected. SPSS software will generate a table of numbers that will be used to randomly assign all eligible patients to the control group (routine treatment) or the Yijinjing group (the modified Yijinjing exercise) at a 1:1 ratio. The intervention will be implemented for 12 weeks. All assessments will be conducted before the intervention, after the intervention, and 3 months after the intervention (see Fig. 1). The schedule for the study is shown in Fig. 2.

### Setting and recruitment

All participants will be recruited at Yueyang Hospital of Integrated Traditional Chinese and Western Medicine and Shanghai Jinhui Rehabilitation Hospital. Both hospitals will post research advertisements to recruit participants. To enhance recruitment quality, the chief investigator will monitor medical records. Recruitment will be carried out by two study researchers, who will carefully record the participants’ past and present medical history.

### Ethical review and general statements

This trial was approved by the Ethics Committee of Yueyang Integrated Hospital of Traditional Chinese and Western Medicine (approval number: 2019-026) and registered in China Clinical Trial Registration Center in October 2019 (registration number: ChiCTR1900026532). All participants will provide written informed consent according to the Helsinki Declaration before participating.

### Eligibility criteria

#### Inclusion criteria

The eligible patients have to meet the following criteria: (1) the clinical diagnosis of stroke according to the Fourth National Academic Conference on Cerebrovascular Diseases Diagnostic Criteria for All Kinds of Cerebrovascular Disease [14] and confirmed by CT or MRI, (2) within 1 year of onset, (3) aged 60–75 years, (4) mild cognition impairment: MoCA< 26 (if the length of schooling is ≤ 12 years, then 1 point will be added to the examination result), (5) balance function: 21 < Berg score < 40, and (6) agreement on participation and signing the informed consent.

#### Exclusion criteria

The exclusion criteria are as follows: (1) patients who are experiencing a severe medical condition, such as heart, liver, kidney, or endocrine diseases; (2) patients with aphasia or hearing impairment; (3) participants of other clinical trials; and (4) patients who are currently using medications to improve cognitive functions.

#### Withdrawal or dropout criteria

1. Participants demands to withdrawal from the study;
2. Participants are diagnosed with other severe disease;
3. Participants showed adverse reactions to the treatment.

### Randomization and allocation concealment

To reduce the chance of bias and increase the probability of meeting statistical analysis assumptions, the chief investigator will receive a computer-generated random list using the statistical software SPSS 24.0 from a statistician independent of the analysis and research teams. Then, the chief investigator will inform the coaches, therapists and participants about the treatment and follow-up plan. Before the first treatment and after the treatment is completed, the assessors will be notified to evaluate all participants.

### Blinding

As this study involves exercise intervention, it is impossible to blind the exercise coaches and participants. To minimize ascertainment bias, the assessors and statistics analyzers will be blinded by two types of blind codes. The allocation result (the Yijinjing exercise group or the routine rehabilitation group) will be showed by using the alphabet “A” or “B” as the first blind code, and then the second blind code represents the real allocation meaning. At the end of the data analysis, the independent research assistant will match the group code “A” or “B” of participants with the real meaning of the group “A” or “B.”

### Intervention

#### Yijinjing exercise group

**Fig. 3:**
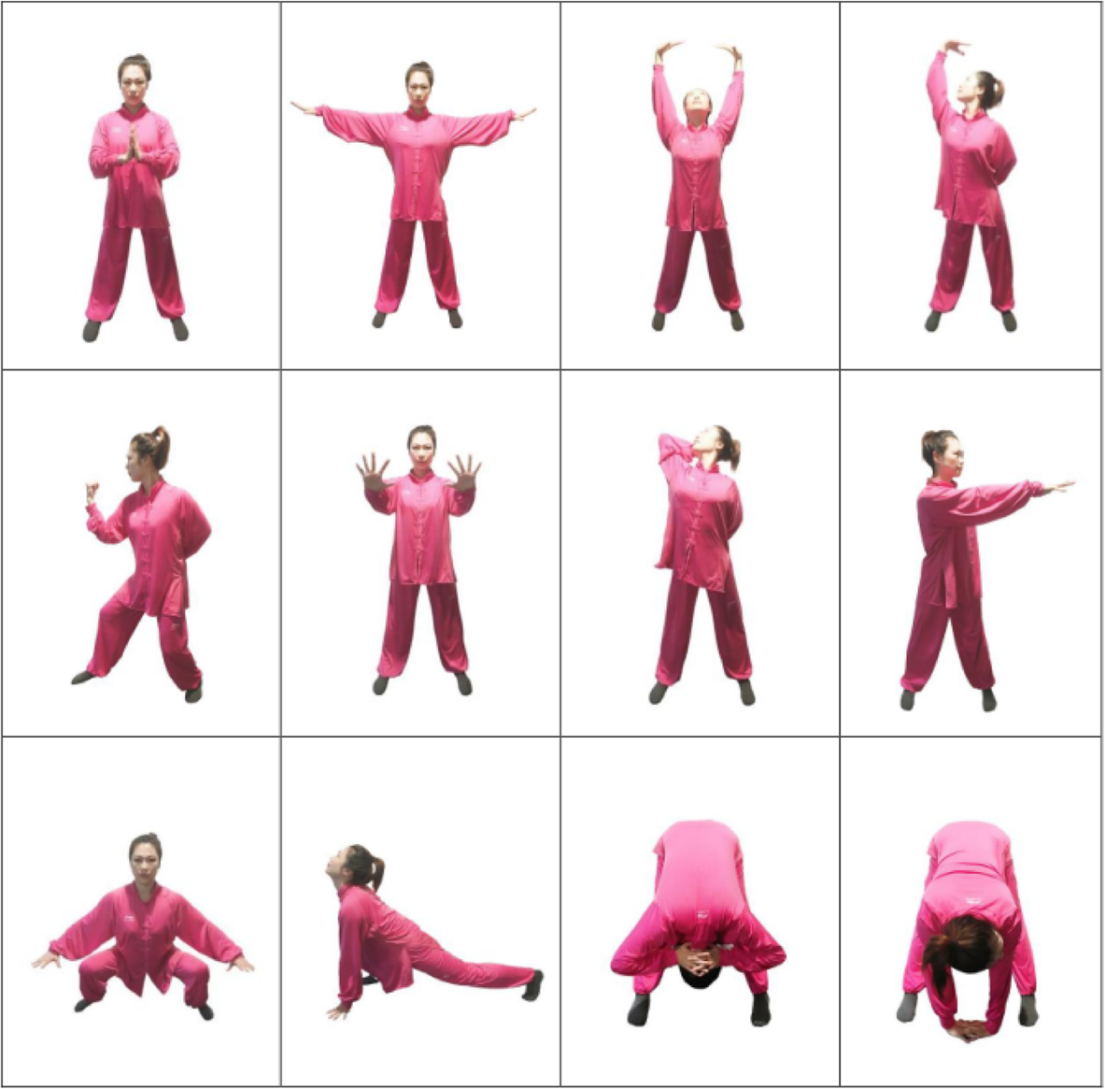
Twelve postures of Yijinjing exercise. The teacher in the image is Pingping Sun, who has agreed to the publication of the images

The participants will receive routine treatment, as well as receive the Yijinjing exercise. The Yijinjing exercise consists of a 12-week Yijinjing practice with 40 min a session, 3 sessions a week. The session has three parts: the warm-up for 5 min, the Yijinjing exercise for 30 min, and the muscle stretching for 5 min. The whole set of the Yijinjing exercise consists of 12 postures (see Fig. 3). Each posture will be demonstrated to the participants, and the Yijinjing exercise is supervised throughout the trial. The same researcher will instruct all participants who will be required to maintain their normal lifestyle, and the postures will be individualized based on their ability.

#### Routine treatment group

With 5 PTs, 5 OTs, and 5 acupuncture physicians providing treatment, the routine treatment group will receive regular rehabilitation services delivered by certified physical therapists, certified occupational therapists, and certified acupuncture physicians, which will be carried out following the inpatient treatment plan. Besides, all participating therapists will be uniformly trained before starting this research to standardize the treatment further. The rehabilitation services include physical therapy (exercise therapy, balance training, joint mobilization), occupational therapy (homework training, hand training, cognitive dysfunction training), acupuncture therapy, and health education.

#### Concurrent treatment

Other cognitive therapies will be not permitted in both two groups, including cognitive stimulation therapy, cognitive-behavioral therapy, and memantine drugs. There are no arrangements for ancillary or post-trial care.

### Outcome measures

#### Primary outcome measures

Montreal Cognitive Assessment-Beijing Version (MoCA): MoCA, a screening tool to assess mild cognitive impairments with a total score of 0–30 [15], will be administered to evaluate global cognition. The assessment domains include visuospatial or executive function, naming, memory, attention, language, abstraction, and delayed memory and orientation [16]. To avoid the learning effect, we will use MoCA 7.1 and MoCA 7.2 for alternate testing.

#### Secondary outcome measures

1. Trail Making Test-A (TMT-A): Studies have shown that executive function is a powerful predictor of disability recovery in patients with acute stroke [17]. Therefore, executive function will be assessed separately using the Trail Making Test-A (TMT-A) [18]. TMT-A is a valid index to assess executive function [19, 20]. The test requires the participants to connect number 1 to 25 sequentially on a piece of paper. The completion time and error numbers will be recorded. Longer time indicates severer cognitive impairment.
2. Chinese vision of the Auditory Verbal Learning Test (AVLT): AVLT will be used to measure memory function, including immediate recall, delayed recall, and recognition. In the immediate recall subtest, participants will be required to repeat after 15 common words immediately after the administrator say the word three times. In the delayed recall subtest, participants will be required to recall the 15 words after 30 min. In the recognition, the tester will be given another 15 unrelated words and 15 words of Part A and required to recognize the original words. Evidence showed that AVLT is a valid assessment tool and more sensitive than the Mini-Mental State Examination (MMSE) and MoCA in assessing memory function [21].
3. Rey Complex Figure Test (RCFT):RCFT will be used to assess visuospatial constructional functions [22]. It uses geometrical figures, including repeated squares, rectangles, triangles, and various other shapes. Participants are asked to copy and then reproduce from memory after 30 min. The total score is 36 points, and the test scores reflect the patients’ visual-construction and motor planning skills.
4. Pittsburgh Sleep Quality Index (PSQI): PSQI will assess the participants’ sleep quality, which consists of 18 self-rated items including 7 domains: subjective sleep quality, sleep latency, sleep duration, habitual sleep efficiency, sleep disturbance, use of sleep medication, and daytime dysfunction over the last month [23]. The participants will answer questions using a 3-point Likert scale. The higher their scores reach, the worse the sleep quality is [24, 25].
5. ODONATE 3D: The ODONATE 3D motion capture, a gait analysis system of Shanghai Maiwo medical technology co. LTD, will assess gait parameters, including the space-time, balance, kinematic, and dynamic parameters. Specific operations are as follows: (1) select the appropriate scene and debug the equipment, (2) set the walking area and guide the patients to complete the walking cycle, (3) the artificial intelligence system captures information, and (4) analyze the captured information.
6. Fugl-Meyer Assessment Scale (FMA)-Motor Function Subtest: FMA Motor Function Subtest will assess motor function. FMA is a three-level cumulative scale to evaluate stroke patients’ sensorimotor recovery with hemiplegia [26]. In this study, the Motor Function Subtest will be used with a total point of 100 (66 points of the upper limb, 34 points of the lower limb). Evidence has shown that FMA is sensitive and reliable [27, 28].
7. Berg Balance Scale (BBS): BBS will assess the balance function by 14 tasks as the quantitative assessment of static and dynamic balance function [29]. BBS is a reliable tool to measure balance function of older adults and disabled people [30, 31], and it can also be used as a risk predictor for accidental falls in stroke patients [32].
8. Modified Barthel Index (MBI): MBL consists of 10 items with 100 points in total [33] and will assess ADL. A higher score indicates a better ability to live independently. Its validity and reliability in evaluation for stroke patients have been reported [34].
9. Short Form 36 Health Survey Questionnaire (SF-36): SF-36, a self-rated assessment, will assess comprehensive self-perceived QOL, such as general health conditions and physical and mental health [35]. Studies have shown high response rates from older stroke patients [36].

### Adherence

All participants in both intervention and control group will be required to log in their activity duration and intensity in a timesheet. The participant will rate the duration and intensity of their activity based on self-perception throughout the day into three levels: low-intensity, moderate-intensity, or high-intensity activities.

### Data management

Data will be collected by assessors using print-based case report forms (p-CRFs). And an identification code will be allocated for each participant to preserve anonymity. During the enrolment phase, two data managers will type the CRF into the computer and check the paper and the electronic data. All study co-applicants and researchers will have access to the final trial dataset. Plans that promote participant retention and complete follow-up are as follows: first, we will strengthen the communication between researchers and participants; second, we will give the participants some “financial incentive”; and third, all treatments and assessments will be free.

### Sample size estimation

Data from our pilot study was utilized to perform the sample size estimation. We used G-Power Software (version 3.1.9.2): F-test, ANOVA: repeated measures with the following parameters: effect size = 0.3; *α* level = 0.05; power (1 − β) = 0.80; group numbers = 2, number of measurements = 3. The total sample size of this study should be a minimum of 62 participants in the two groups. With a 15% attrition rate considered, the sample size of 72 participants is necessary.

### Statistical analysis

Statistical analyses will be carried out using SPSS software version 24.0 (SPSS, Inc., Chicago, IL, USA) and Microsoft Excel 2016 software. Data will be presented as means, standard deviations, and percentage changes. The intention to treat (ITT) principle will be used for statistical analysis, and the multiple imputation will reduce the missing data. Repeated measures ANCOVA will be applied for each measure to compare test performance between the Yijinjing exercise and routine treatment, with baseline scores used as covariates. Statistical significance will be considered at *p* value < 0.05.

### Safety and adverse events

If the patient has been randomized, adverse events (AE) monitoring will begin until the end of this study. Researchers will record any abnormalities on the CRF including dizziness, dyspnea, and fatigue and assess reported AEs for severity and relatedness to the intervention. All participants will also be instructed to report any other adverse effects to the researchers whenever they occur. Researchers will try their best to prevent possible AEs. In case of any discomfort during the participation, the participants will receive treatment and corresponding financial compensation. Serious AEs will be reported to the ethics committee, coordinating center, steering committee, and sponsor immediately.

### Organization

Members of the coordinating center include the director, information manager, database programmer, statistical analyst, project coordinator, and research assistants. Members of the steering committee include the study chair, data coordinating center director, statistician, and clinical center directors. In addition, the responsibilities of the endpoint adjudication committee are included in the ethics committee and the data management team is included in the coordinating center. Therefore, there is no independent endpoint adjudication committee and data management team.

### Dissemination

Results will be freely disseminated via a peer-reviewed report to the funder (Shanghai University of Traditional Chinese Medicine) and through open access journal articles and conference presentations. In addition, we will inform all participants of the results through a brief report. There will be no use of professional writers. Public or professional access to the full protocol, participant-level data and statistical code will be by request to the chief investigator.

## Discussion

As a traditional Chinese exercise, Yijinjing was developed based on the traditional Chinese Medicine theory and is related to harmonizing Yin and Yang, which connects the body with the mind to improve physical and cognitive functions. Most of the previous studies focus on the effects of traditional Chinese exercises on healthy older people. Therefore, this research is the first randomized controlled trial that evaluates the effectiveness of the Yijinjing practice for PSCI patients. We expect that Yijinjing will become an innovative and effective treatment approach to improve patients’ cognitive functions with PSCI and expect it to show superior effects compared with the control group. We also expect the study’s outcome to provide evidence for clinical workers and researchers working with post-stroke populations.

In the outcome measure stage, we will use multiple assessment tools to comprehensively evaluate the cognition domains ranging from global cognitive function to specific cognitive domains, such as memory, executive function, and visuospatial functions. However, this study still has some limitations. First, the insufficient sample size and short intervention time could be potential factors that affect the study outcomes. Next, treatment may likely be more successful involving interventions with individuals who have a robust home-support network. This research’s recruitment method may be more inclined to patients with strong family support, which will lead to a conclusion that may not apply to all stroke patients. Besides, this exercise may not be appropriate to the participants who have a low level of motor functions or no movement in the body’s affected side.

In summary, this study’s main objective is to investigate Yijinjing’s effectiveness in improving PSCI patients’ cognitive functions and explore the superior outcomes of Yijinjing compared with conventional rehabilitation therapy.

### Trial status

The study registration number is ChiCTR1900026532. The recruitment is in progress. Recruitment began on May 2019 and will be completed by May 2021.

## Data Availability

The data and the relevant results in this study will be shared through the scientific papers.

## Abbreviations

PSCI: Post-stroke cognitive impairment
RCT: Randomized controlled trial
QOL: Quality of life
MoCA: Montreal Cognitive Assessment
TMT-A: Trail Making Test-A
AVLT: Auditory Verbal Learning Test
CFT: Complex Figure Test
PSQI: Pittsburgh Sleep Quality Index
FMA: The Fugl-Meyer Assessment Scale
BBS: The Berg Balance Scale
ADL: Activities of daily living
MBI: The modified Barthel Index
SF-36: The Short Form 36 Health Survey Questionnaire

## References

[1] Mcentire CRS, Choudhury GR, Torres A (2016). Impaired arm function and finger dexterity in a nonhuman primate model of stroke. Stroke.

[2] Yang S, Ye H, Huang J, Tao J, Jiang C, Lin Z, Zheng G, Chen L (2014). The synergistic effect of acupuncture and computer-based cognitive training on post-stroke cognitive dysfunction: a study protocol for a randomized controlled trial of 2×2 factorial design. BMC Complement Altern Med.

[3] Adamit T, Maeir A, Ben Assayag E, Bornstein NM, Korczyn AD, Katz N (2015). Impact of first-ever mild stroke on participation at 3 and 6 month post-event: the TABASCO study. Disabil Rehabil.

[4] Poynter L, Kwan J, Vassallo M (2013). How does cognitive impairment impact on functional improvement following the rehabilitation of elderly patients?. Int J Clin Pract.

[5] Lo Coco D, Lopez G, Corrao S (2016). Cognitive impairment and stroke in elderly patients. Vasc Health Risk Manag.

[6] Kavirajan H, Schneider LS (2007). Efficacy and adverse effects of cholinesterase inhibitors and memantine in vascular dementia: a meta-analysis of randomised controlled trials. Lancet Neurol.

[7] Jiao L, Zhang J, Li Z, Liu H, Chen Y, Xu S (2011). Edaravone alleviates delayed neuronal death and long-dated cognitive dysfunction of hippocampus after transient focal ischemia in Wistar rat brains. Neuroscience.

[8] Tomassoni D, Lanari A, Silvestrelli G, Traini E, Amenta F (2008). Nimodipine and its use in cerebrovascular disease: evidence from recent preclinical and controlled clinical studies. Clin Exp Hypertens.

[9] Johansson BB (2004). Brain plasticity in health and disease. Keio J Med.

[10] Han P, Zhang W, Kang L (2017). Clinical evidence of exercise benefits for stroke. Exercise for cardiovascular disease prevention and treatment.

[11] Vanderbeken I, Kerckhofs E (2017). A systematic review of the effect of physical exercise on cognition in stroke and traumatic brain injury patients. Neuro Rehabil.

[12] Chen CH, Hung KS, Chung YC, Yeh ML (2019). Mind–body interactive qigong improves physical and mental aspects of quality of life in inpatients with stroke: a randomized control study. Eur J Cardiovasc Nurs.

[13] Zou L, Yeung A, Zeng N, Wang C, Sun L, Thomas G, Wang H (2018). Effects of mind-body exercises for mood and functional capabilities in patients with stroke: an analytical review of randomized controlled trials. Int J Environ Res Public Health.

[14] The Fourth Academic Seminar of the Chinese Society for Neuroscience (1996). Major diagnostic points of cerebrovascular disease. Chin J Neurol.

[15] Li X, Jia S, Zhou Z, Jin Y, Zhang X, Hou C, Zheng W, Rong P, Jiao J (2018). The role of the Montreal Cognitive Assessment (MoCA) and its memory tasks for detecting mild cognitive impairment. Neurol Sci.

[16] Shaik MA, Chan QL, Xu J, Xu X, Hui RJY, Chong SST, Chen CLH, Dong YH (2016). Risk factors of cognitive impairment and brief cognitive tests to predict cognitive performance determined by a formal neuropsychological evaluation of primary health care patients. J Am Med Dir Assoc.

[17] Park YH, Jang J-W, Park SY, Wang MJ, Lim J-S, Baek MJ, Kim BJ, Han M-K, Bae H-J, Ahn S, Kim SY (2015). Executive function as a strong predictor of recovery from disability in patients with acute stroke: a preliminary study. J Stroke Cerebrovasc Dis.

[18] Tombaugh TN (2004). Trail Making Test A and B: normative data stratified by age and education. Arch Clin Neuropsychol.

[19] Fällman K, Lundgren L, Wressle E (2019). Normative data for the oldest old: Trail Making Test A, Symbol Digit Modalities Test, Victoria Stroop Test and Parallel Serial Mental Operations. Aging Neuropsychol Cogn.

[20] Laere E, Tee SF, Tang PY (2018). Assessment of cognition in schizophrenia using trail making test: a meta-analysis. Psychiatry Investig.

[21] Harris ME, Ivnik RJ, Smith GE (2002). Mayo’s older Americans normative studies: expanded AVLT recognition trial norms for ages 57 to 98. J Clin Exp Neuropsychol.

[22] Delaney RC, Prevey ML, Cramer J, Mattson RH, VA Epilepsy Cooperative Study #264 Research Group (1992). Test-retest comparability and control subject data for the Rey-auditory verbal learning test and Rey-Osterrieth/Taylor complex figures. Arch Clin Neuropsychol.

[23] Buysse DJ, Reynolds CF, Monk TH, Berman SR, Kupfer DJ (1989). The Pittsburgh Sleep Quality Index: a new instrument for psychiatric practice and research. Psychiatry Res.

[24] Hinz A, Glaesmer H, Brähler E, Löffler M, Engel C, Enzenbach C, Hegerl U, Sander C (2017). Sleep quality in the general population: psychometric properties of the Pittsburgh Sleep Quality Index, derived from a German community sample of 9284 people. Sleep Med.

[25] Spira AP, Beaudreau SA, Stone KL (2012). Reliability and validity of the Pittsburgh Sleep Quality Index and the Epworth Sleepiness Scale in older men. J Gerontol Ser A Biomed Sci Med Sci.

[26] Fugl-Meyer AR, Jääskö L, Leyman I, Olsson S, Steglind S (1975). The post-stroke hemiplegic patient. 1. a method for evaluation of physical performance. Scand J Rehabil Med.

[27] Hernández ED, Galeano CP, Barbosa NE (2019). Intra-and inter-rater reliability of fugl-meyer assessment of upper extremity in stroke. J Rehabil Med.

[28] Rech KD, Salazar AP, Marchese RR, Schifino G, Cimolin V, Pagnussat AS (2020). Fugl-Meyer assessment scores are related with kinematic measures in people with chronic hemiparesis after stroke. J Stroke Cerebrovasc Dis.

[29] Berg K, Wood-Dauphine S, Williams JI (1989). Measuring balance in the elderly: preliminary development of an instrument. Physiother Can.

[30] Blum L, Korner-Bitensky N (2008). Usefulness of the Berg Balance Scale in stroke rehabilitation: a systematic review. Phys Ther.

[31] Downs S (2015). The Berg Balance Scale. J Physiother.

[32] Lajoie Y, Gallagher SP (2004). Predicting falls within the elderly community: comparison of postural sway, reaction time, the Berg balance scale and the Activities-specific Balance Confidence (ABC) scale for comparing fallers and non-fallers. Arch Gerontol Geriatr.

[33] Leung SO, Chan CC, Shah S (2007). Development of a Chinese version of the Modified Barthel Index-- validity and reliability. Clin Rehabil.

[34] Ohura T, Hase K, Nakajima Y, Nakayama T (2017). Validity and reliability of a performance evaluation tool based on the modified Barthel Index for stroke patients. BMC Med Res Methodol.

[35] Jenkinson C, Coulter A, Wright L (1993). Short form 36 (SF36) health survey questionnaire: normative data for adults of working age. BMJ.

[36] O'Mahony PG, Rodgers H, Thomson RG (1998). Is the SF-36 suitable for assessing health status of older stroke patients?. Age Ageing.

